# Combined Effects of Gold Nanoparticles and Ionizing Radiation on Human Prostate and Lung Cancer Cell Migration

**DOI:** 10.3390/ijms20184488

**Published:** 2019-09-11

**Authors:** Elham Shahhoseini, Bryce N. Feltis, Masao Nakayama, Terrence J. Piva, Dodie Pouniotis, Salem S. Alghamdi, Moshi Geso

**Affiliations:** 1 Discipline of Medical Radiation, School of Health and Biomedical Sciences, RMIT University, Bundoora 3083, Victoria, Australia; elham.shahhoseini@rmit.edu.au (E.S.); masao.nakayama@rmit.edu.au (M.N.); 2 Discipline of Human Bioscience, School of Health and Biomedical Sciences, RMIT University, Bundoora 3083, Victoria, Australia; bryce.feltis@rmit.edu.au (B.N.F.); terry.piva@rmit.edu.au (T.J.P.); 3 Discipline of Laboratory Medicine, School of Health and Biomedical Sciences, RMIT University, Bundoora 3083, Victoria, Australia; dodie.pouniotis@rmit.edu.au; 4 Department of Radiological Sciences, Collage of Applied Medical Science, Imam Abdulrahman Bin Faisal University, Dammam 34212, Saudi Arabia; salemghamdi@hotmail.com

**Keywords:** cell migration, gold nanoparticles, ionizing radiations, cell viability, cell adherence

## Abstract

The effect of 15 nm-sized gold nanoparticles (AuNPs) and/or ionizing radiation (IR) on the migration and adhesion of human prostate (DU145) and lung (A549) cancer cell lines was investigated. Cell migration was measured by observing the closing of a gap created by a pipette tip on cell monolayers grown in 6-well plates. The ratio of the gap areas at 0 h and 24 h were used to calculate the relative migration. The relative migration of cells irradiated with 5 Gy was found to be 89% and 86% for DU145 and A549 cells respectively. When the cells were treated with 1 mM AuNPs this fell to ~75% for both cell lines. However, when the cells were treated with both AuNPs and IR an additive effect was seen, as the relative migration rate fell to ~60%. Of interest was that when the cells were exposed to either 2 or 5 Gy IR, their ability to adhere to the surface of a polystyrene culture plate was significantly enhanced, unlike that seen for AuNPs. The delays in gap filling (cell migration) in cells treated with IR and/or AuNPs can be attributed to cellular changes which also may have altered cell motility. In addition, changes in the cytoskeleton of the cancer cells may have also affected adhesiveness and thus the cancer cell’s motility response to IR.

## 1. Introduction

Ionizing radiation (IR) is used to kill cancer cells which forms the basis of radiation therapy [1]. One of the main challenges in clinical radiation oncology is cancer metastasis and the formation of secondary deposits at sites distant from the original tumour [2]. Cancer metastases are formed when cells migrate from the primary tumour site to other regions in the body [3,4,5]. Due to the importance of cell migration and its role in metastasis, the effect of IR on this process has been investigated under both in vivo and in vitro conditions but contradictory effects have been reported [6]. Several studies have demonstrated that IR might enhance cell migration when cells are irradiated with low lineal energy transfer (LET) radiation such as X-rays but can be inhibiting when such cells are exposed to high LET radiation e.g., carbon-ion beams [7,8,9,10]. However, some studies have shown that both high and low LET radiation slowed the rate of A549 lung cancer cell migration [11,12]. Different molecular mechanisms have been suggested to explain the effect IR has on cancer cell migration, that (a) it enhances epithelial-mesenchymal transition (EMT) and as a result cell migration [13], (b) modifies matrix metalloproteinases (MMP) which results in the degradation of the extracellular matrix (ECM) which promotes cell migration [14,15], and (c) high LET radiation decreases expression of integrins which inhibits cell migration [16,17]. Also, low LET X-rays have been shown to increase cell-ECM adhesion [18] which could diminish the migration of some cancer cells.

Since, radiation therapy increases local tumour control and overall survival [19], it is difficult to validate the pre-clinical (in vitro and in vivo) results of the effects of IR on cancer cell migration/metastasis. Further research is needed to better identify the actual mechanism of cell migration that occurs following exposure to IR.

Another external source/stimuli that has been investigated regarding the effects of IR on cell migration are nanoparticles (NPs). Gold nanoparticles (AuNPs) have been found to be bio-compatible [20] and their toxicity shown to be size- and concentration-dependent [21]. Different types of nano-gold such as gold nano-rods (AuNRs) or gold-platinum nano-seeds (Au@Pt-Nss), were reported to have inhibitory effects on cancer cell migration. AuNRs attenuate the migration and invasion of cancer cells by impairing the actin cytoskeletal assembly, decreasing mitochondrial oxidative phosphorylation and decreasing ATP synthesis [22] while Au@Pt-Nss decreased cell migration by suppressing MMP expression [23]. Very few studies have investigated the effects of AuNPs on cancer cell migration. AuNPs with no surface modification have been shown to inhibit cell migration by affecting focal adhesion kinase (FAK) and MMP-2 activity [24]. In a recent in vitro study, 10 nm AuNPs suppressed papillary thyroid cancer (PTC) cell migration through downgrading CCT3 gene transcription [25].

Despite extensive studies on the combination of AuNPs with different types of IR in potential cancer treatments [26], very little is known about the combined effects that AuNPs and IR may have on cell migration.

In this study, the effect of 15 nm spherical AuNPs on the cell motility and migration of human prostate (DU145) and lung (A549) cancer cells was investigated. The experiments were performed with and without IR and to determine if up to 5 Gy IR (X-rays) enhanced any motility effects. Radiation doses of 2 and 5 Gy were chosen because these are generally employed as a single fraction dose in many patient’s radiation treatment regimens. The results showed that both AuNPs and IR individually reduced the migration of human prostate (DU145) and lung (A549) cancer cells, and in combination they elicited an additive effect. In addition, due to the important role of cell adherence in cell migration, the effect of IR and AuNPs on prostate (DU145) and lung (A549) cancer cells was examined and the results showed that the former but not the latter enhanced the adherence of these cells.

## 2. Results

### 2.1. Cellular Uptake of Gold Nanoparticles (AuNPs)

**Figure 1:**
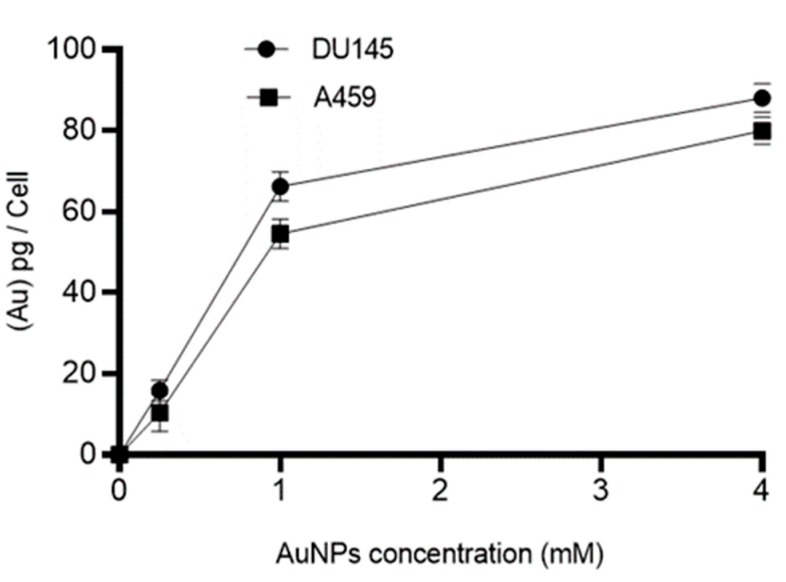
Inductively coupled plasma mass spectrometry (ICP MS) results of cellular uptake of gold nanoparticles (AuNPs) in human cancer cells. The ICP MS intensities were converted to mass of AuNPs per cell by using Au ion standard curve and cell count. No AuNPs were detected in control cells. Results are expressed mean ± standard error of the mean (SEM) of 3 replicates.

The mass of Au in picograms (pg) per cell for human prostate (DU145) and lung (A549) cancer cell was measured using inductively coupled plasma mass spectrometry (ICP MS). The cells were treated with different concentrations (control (0), 0.25, 1, and 4 mM) of AuNPs for 24 h prior to ICP MS measurement. As shown in Figure 1, no gold was detected in control groups whilst the amount of gold in treated groups increased in a concentration-dependent manner. Cellular uptake of 15 nm AuNPs by DU145 and A549 approached saturation at 1 mM and, based on these results, this concentration (1 mM) was chosen for subsequent experiments.

### 2.2. The Effects of AuNPs on Cell Viability 

**Figure 2:**
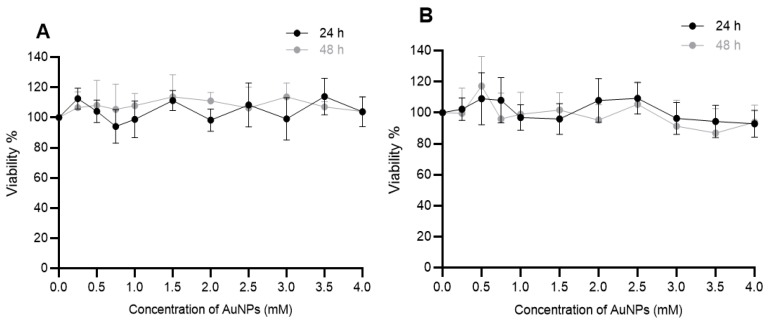
Effect of AuNPs on the viability of human cancer cells in vitro. (**A**) DU145 (prostate) and (**B**) A549 (lung) cancer cells were treated with AuNPs for either 24 and/or 48 h and cell viability (viability%) was determined using the MTS assay. Results expressed as the mean ± SEM of 3 replicates.

The effect of AuNPs on the viability of DU145 and A549 cancer cell was determined by exposing the cells to different concentrations (0–4 mM) of AuNPs for 24 and 48 h. Cell viability was measured using the (3-(4,5-dimethylthiazol-2-yl)-5-(3-carboxymethoxyphenyl)-2-(4-sulfophenyl)-2H-tetrazolium, inner salt) MTS assay and the results shown in Figure 2. It can be seen that the 15 nm AuNPs even at 4 mM had no negative impacts on the viability of both cells over 48 h.

### 2.3. The Effects of Ionizing Radiation (IR) on Cell Viability

**Figure 3:**
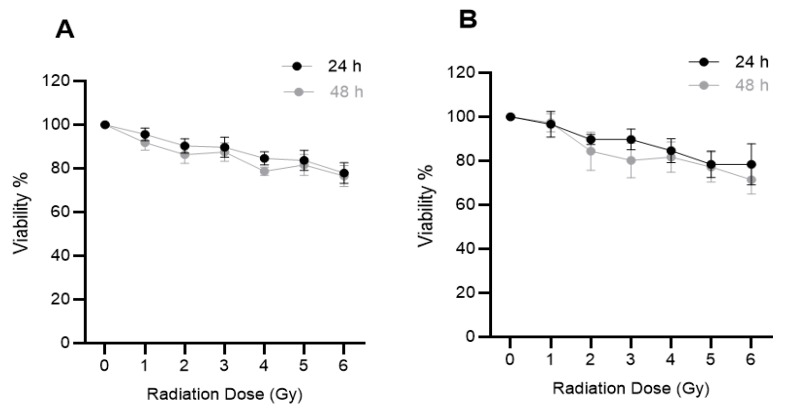
Effect of ionizing radiation (IR) on the viability of human cancer cells in vitro. (**A**) DU145 (prostate) and (**B**) A549 (lung) cancer cells were irradiated with 0–6 Gy X-rays and cell viability (viability %) was determined after 24 h or 48 h using the MTS assay. Results expressed as the mean ± SEM of 3 replicates.

The effect of IR on the viability of prostate and lung cancer cells was determined by exposing DU145 and A549 cells to different doses (0–6 Gy) of 6 MV X-rays. Cell viability was measured at 24 and 48 h post-irradiation using the MTS assay. At 6 Gy cell viability fell to ~80% after 48 h post-exposure for both cell lines (Figure 3). From these results, 2 and 5 Gy were chosen as the radiation dose used in the migration and cell adhesion studies. These dose ranges were chosen based on the standard dose fractionation regimen that is commonly used in radiation therapy [27].

### 2.4. The Effects of Combination of Ionizing Radiation (IR) and AuNPs on Cell Viability

**Figure 4:**
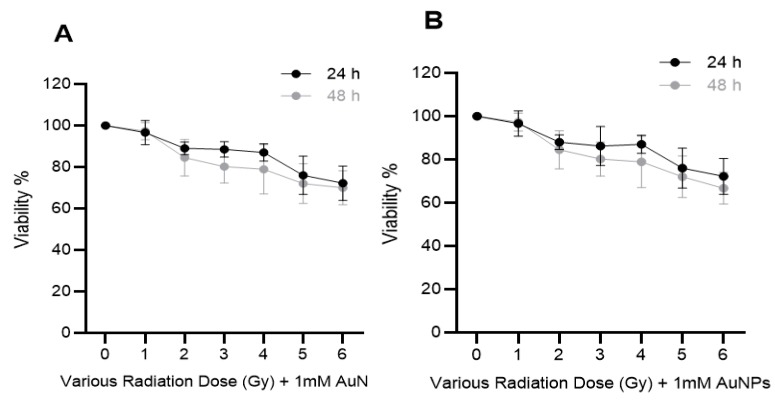
Effect of combination of IR and AuNPs on the viability of human cancer cells in vitro. (**A**) DU145 (prostate) and (**B**) A549 (lung) cancer cells were treated with 1 mM AuNPs for 24 h prior to exposure to 0–6 Gy of 6 MV X-rays and cell viability (viability %) was measured after 24 h or 48 h post-irradiation using the MTS assay. Results expressed as the mean ± SEM of 3 replicates.

The effect of IR and/or AuNPs on DU145 and A549 cell viability was examined by treating the cells with 1 mM AuNPs 24 h prior to being exposed to different doses (0–6 Gy) of 6 MV X-rays. Cell viability was measured 24 or 48 h post-irradiation using the MTS assay (Figure 4). It can be seen that AuNPs did not increase the cytotoxic effects of IR on either cell type.

### 2.5. The Additive Effect of IR and AuNPs on Cell Migration

**Figure 5:**
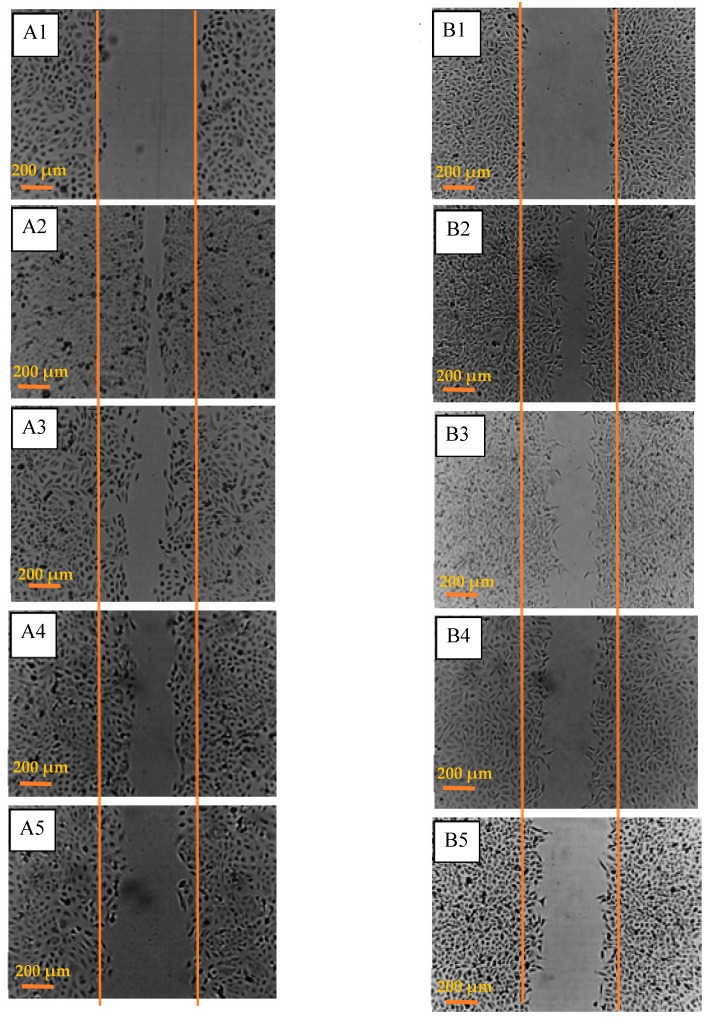
Effect of IR and/or AuNPs on cell motility in vitro. (**A**) DU145 (prostate) cancer cells; (**A1**) untreated controls at 0 h, (**A2**) untreated control at 24 h, (**A3**) cells exposed to 5 Gy X-rays after 24 h, (**A4**) cells treated with 1 mM AuNPs at 24 h and, (**A5**) cells treated with IR and AuNPs at 24 h and, (**B**) lung (A549) cancer cells; (**B1**) untreated controls at 0 h, (**B2**) untreated control at 24 h, (**B3**) cells exposed to 5 Gy X-rays after 24 h, (**B4**) cells treated with 1 mM AuNPs at 24 h and, (**B5**) cells treated with IR and AuNPs at 24 h.

Scratch assays were performed to assess the effects of 5 Gy irradiation and/or 1 mM AuNPs on cell migration in vitro. A 200 µL sterile pipette tip was used to make a gap in confluent monolayers of DU145 and A549 cells. The migration of the treated cells into the denuded area (marked with orange lines) was observed over 24 h (Figure 5).

**Figure 6:**
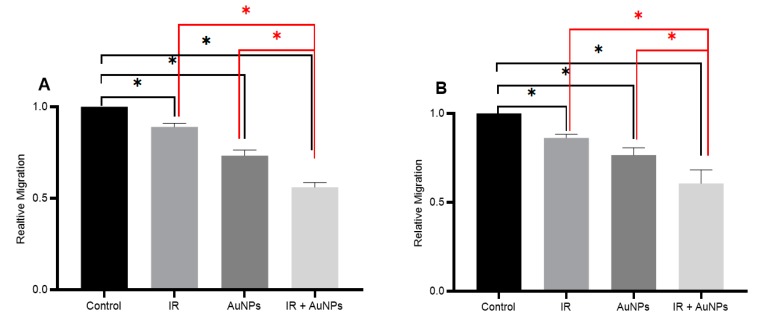
The effect of IR and/or AuNPs on human cancer cell migration in vitro. Relative cell migration of (**A**) DU145 (prostate) and, (**B**) A549 (lung) cancer cells. Control represents untreated cells, IR: cells irradiated with 5 Gy X-ray, AuNPs: cells treated with 1 mM AuNPs, and IR+ AuNPs: cells were treated with both 5 Gy X-ray and 1 mM AuNPs. Results expressed as the mean ± SEM of 3 replicates. Significance of different treatments compared to control is represented by black lines, and between treatments is represented by red lines is shown as * *p* < 0.05.

Cells treated with only IR showed a ~15% decrease in the gap closure rate compared to their respective untreated controls (Figure 6). When both cell lines were exposed to AuNPs for 24 h prior to creating the 600 µm gap, they migrated ~25% slower than that of untreated controls (Figure 6). When these cells were treated with both IR and AuNPs there was a 40% reduction in gap closure which showed the additive effects of both treatments.

**Figure 7:**
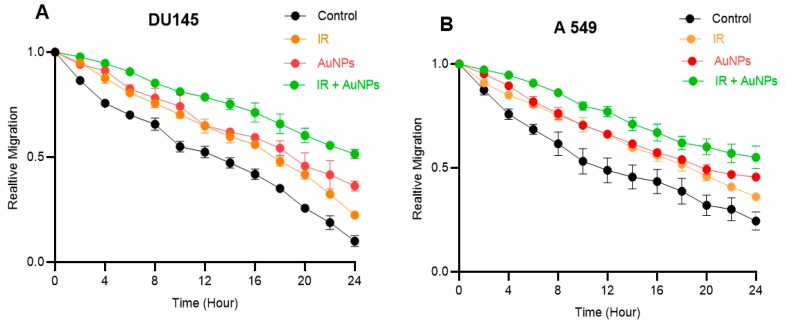
Effect of IR and/or AuNPs on the relative migration of DU145 and A549 cells in vitro. (**A**) DU145 (prostate) and (**B**) A549 (lung) cancer cells; the first 6 h is marked with the circle. Results expressed as the mean ± SEM of 3 replicates.

Gap closure was recorded using a time-lapse camera and the area of the gap was measured every 2 h. The relative migration over 24 h for untreated (control) and treated DU145 and A549 cells are shown in Figure 7. The highest relative migration rate was observed for untreated controls while the lowest was seen in cells treated with both IR and AuNPs. A linear regression line was fitted on each curve and the slope of the fitted line was considered as the gap filling rate.

There was a difference in gap filling rates between the untreated control and treated groups in the first 6 h following gap creation (marked with the black circle on Figure 7).

Within the first 6 h, the gap filling rate of untreated control cells was faster compared to the treated groups. However, after 8 h, the gap filling rates observed in both untreated and treated groups in both cell types were similar. The details of the gap filling rate in each time range for both cell types are tabulated in Table 1.

As seen in Table 1, the gap filling rate in the untreated controls for both cell lines follow a mixed pattern [meaning that the filling rate (during the first 6 h) begins with faster rate e.g., −0.050 and −0.053 for DU145 and A549 cells, respectively, and then continues at a slower rate for the subsequent 18 h e.g., −0.033 for DU145 and −0.021 for A549 cells].

Treating the cells with either IR and/or AuNPs affects this pattern and slows down the gap filling rate in a way that there is no significant difference between the rates observed during the first 6 h compared to that seen for the next 18 h.

### 2.6. The Effect of IR on Cell Adhesion

**Figure 8:**
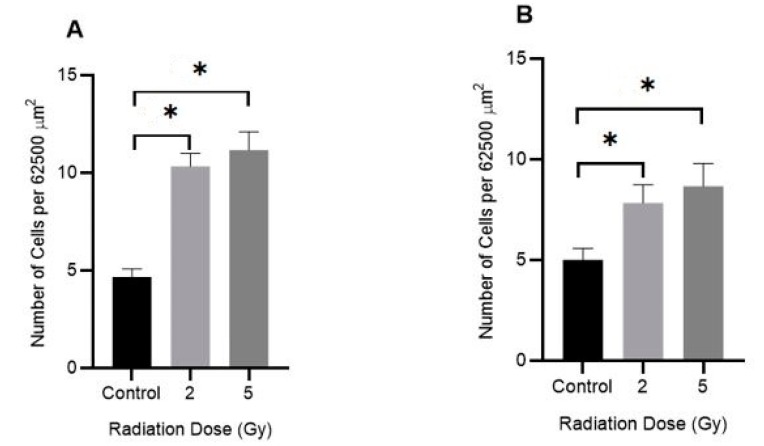
Effect of IR on the adhesion of human cancer cells in vitro. (**A**) DU145 (prostate) and (**B**) A549 (lung) cancer cells were exposed to either 2 or 5 Gy (6 MV X-rays) and after 24 h the cells were trypsinised and plated in 6-well plates. After 4 h, the number of adhered cells in a 62,500 μm2 area were counted. Results are expressed as mean ± SEM of 3 replicates. Significance of different IR doses on cell adherence is shown as * *p* < 0.05.

The effect of IR on the adhesiveness of DU145 and A549 cancer cell lines were measured using a microscopy and imaging-based adhesion assay. Adherent cells grown in tissue culture flasks were exposed to either 2 or 5 Gy of 6 MV X-rays and after 24 h, they were trypsinised and cells plated out into a 6-well plate. After 4 h incubation, the wells were gently washed with phosphate-buffered saline (PBS) and the number of attached cells in a defined area (0.25 × 0.25 mm or 62,500 μm^2^) was counted (Figure 8). Exposure to IR enhanced the adhesiveness of both tumour cell lines by ~100%.

### 2.7. The Effect of AuNPs on Cell Adhesion

**Figure 9:**
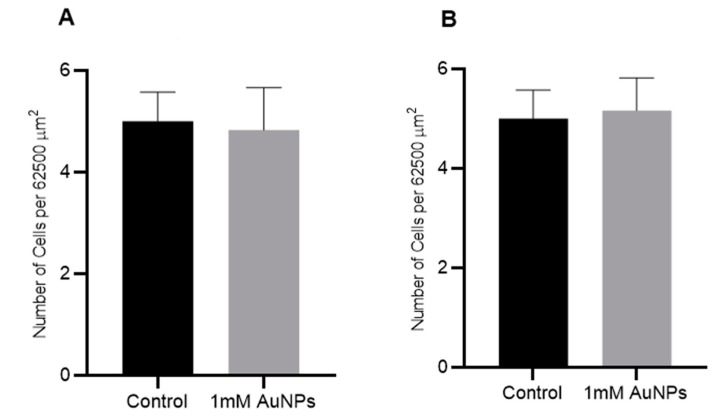
Effect of AuNPs on cell adhesion of human cancer cells in vitro. (**A**) DU145 (prostate) and (**B**) A549 (lung) cancer cells were treated with 1 mM AuNPs and after 24 h the cells were trypsinised and plated in 6 well plates. After 4 h, the number of adhered cells in a 62,500 μm^2^ area were counted. Results are expressed mean ± SEM of 3 replicates.

The effect of 1 mM AuNPs on the adhesiveness of DU145 and A549 cancer cell lines was measured using a microscopy and imaging-based adhesion assay (Figure 9). Adherent cells grown in tissue culture flasks were treated with 1 mM AuNPs and, after 24 h, they were trypsinised and cells plated out into the wells of a 6-well plate. After 4 h incubation, the plates were gently washed with PBS and the number of attached cells in a defined area (0.25 × 0.25 mm or 62,500 μm^2^) were counted. Treatment of the cells with 1 mM AuNPs had no noticeable effect on cell adhesion.

### 2.8. The Effects of a Combination of IR and AuNPs on Cell Adhesion

**Figure 10:**
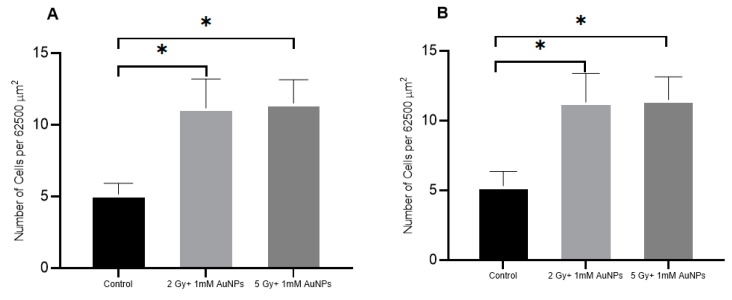
Effect of IR and/or AuNPs on the adhesion of human cancer cells in vitro. (**A**) DU145 (prostate) and (**B**) A549 (lung) cancer cells treated with 1 mM AuNPs 24 h prior to exposure to either 2 or 5 Gy (6 MV X-rays) and after 24 h the cells were trypsinised and plated in 6 well plates. After 4 h, the number of adhered cells in a 62,500 μm2 area were counted. Results are expressed mean ± SEM of 3 replicates. Significance of different IR doses with AuNPs on cell adherence compared to untreated controls is shown as * *p* < 0.05.

The combined effect of IR and/or AuNPs on DU145 and A549 cell adhesiveness was examined by treating the cells with 1 mM AuNPs 24 h prior to being exposed to two different doses (2 and 5 Gy) of 6 MV X-rays. As shown in Figure 10 the combination of AuNPs and IR did not increase the cell adhesiveness when compared to cells treated with IR alone (Figure 8).

## 3. Discussion and Conclusion

Metastasis or the ability of tumour cells to invade other remote organs remains a challenge in the treatment of cancers and many different modalities have been applied to attempt to prevent this from occurring. Despite contradictory reports on the effects of IR on cancer cell migration [6], a few studies have shown a reduction in the rate of cell migration [11,12]. Panzetta et al. has also shown that IR increases SV40 cancer cell-ECM adhesion by decreasing paxillin and integrin levels in the cytoskeleton [18] which inhibits cell motility.

In this study, the initial cell migration assays were performed to evaluate the effects of IR on cancer cell migration. These results showed that 5 Gy of X-rays retards prostate and lung cancer cell migration by 11–14%.

Since, our results showed an inhibitory effect of IR on cancer cell migration, and as it has been reported [24,25] that AuNPs also retard cancer cell migration, we examined the effects of IR and/or AuNPs on prostate (DU145) and lung (A549) cancer cell migration. Our results clearly showed that both IR and AuNPs, individually and in combination, have a negative impact on the motility of both DU145 and A549 cells. In fact, the highest inhibitory effects (25%) were seen in cells treated with both IR and AuNPs.

We also examined the effects of IR on prostate and lung cancer cell adhesiveness and our results showed that both cell types become more adherent (in a dose-dependent manner) when they were exposed to either a 2 or 5 Gy dose of 6 MV X-rays (Figure 8). We postulate that this effect may have been caused by several factors. The interaction of IR with the cell culture media (mainly water molecules) can lead to water radiolysis, resulting in the formation of a large number of secondary electrons. Secondary electrons are the result of the atomic interactions of the incident X-rays via both photoelectric and Compton effects [27]. The water molecules through a chain reaction with some secondary electrons are converted to different types of reactive oxygen species (ROS) such as hydrogen peroxide (H_2_O_2_) [28] and it has been reported [29] that some types of ROS, i.e., superoxide onions (O^ˉ^) and H_2_O_2_, can inhibit cancer cell migration and invasion. It is possible that DNA damage directly caused by IR may also retard cell migration. However, further studies are needed to confirm the mechanism by which IR exerts this effect. The 2 and 5 Gy IR dose were chosen as they are similar to the doses used in standard dose fractionation in radiotherapy [30]. Our results are consistent with that of Panzetta et al. [18] which suggests that IR are affecting cell adhesiveness by a similar mechanism. Of interest was that no significant change in cell adhesion was observed when these cells were treated with 1 mM AuNPs alone (Figure 9).

The viability results for cells treated with AuNPs showed that after 48 h, 1 mM AuNPs had no negative effect on cell viability which suggests that the slower cell migration in treated groups is not due to changes in the rate of cell death. However, cells irradiated with IR showed a ~3% (for 2 Gy) and ~15% (for 5 Gy) decrease in viability. To minimise the effects of damaged cells on cell migration, cells were incubated for 24 h post-radiation and the culture media was changed before the scratch test was performed. Therefore, by excluding the damaged/dead cells, only functional cells were involved in migration assay.

Upon closer observation of the gap filling rate (Figure 7), it can be seen that the untreated (control) cells filled the gap at a faster starting rate for the first 6 h, after which they slowed for the next 18 h. However, the migration rates in treated cells were found to be slower from the beginning i.e., 0 h and no significant difference in the slopes of the curves between treated cells were observed before and after 6 h (Table 1). This effect is clearly a result of the reaction of the treated cells to the external stimuli i.e., IR and/or AuNPs. However, the detailed mechanism for these variations requires further investigations which is beyond the scope of this research.

The chief aim of this work was to study the individual and combined effects of IR and AuNPs on cancer cell motility. Both treatments either alone or in combination retarded the movement of human prostate and lung cancer cells. The highest inhibitory effects on cancer cell migration was seen with the combination of IR and AuNPs. In addition, our study on the effects of IR and AuNPs on cancer cell adhesiveness showed that IR increased cell adhesiveness while AuNPs had no effect. This research needs to be extended to include other cancer types so see if the effects seen here for IR and/or AuNPs are consistent across a variety of tumour cell types. Likewise, it is also crucial to undertake this study on normal primary cells to observe if they also behave in a similar fashion to that of their tumorous derivatives.

This research represents the initial steps of a broader study based on investigation into the effects of external stimuli (IR and AuNPs) on cellular biomechanics, motility and adhesiveness. further work will include untransfected primary cells and their responses will be validated against tumour cells of the same type. The outcomes of these studies will have potential impacts on future radiation dose delivery methodologies and cancer treatments via radiotherapy.

## 4. Materials and Methods

### 4.1. Inductively Coupled Plasma Mass Spectrometry (ICP MS) Measurment of Celluar AuNPs

In order to determine the uptake of AuNPs by the cells, ICP MS (Agilent 7700, Santa Clara, CA, USA) measurements were performed. Human prostate cancer (DU145) and lung cancer (A549) cells were seeded (10^6^ cells/well), in 6 well plates then incubated at 37 °C with 5% CO_2_ in a humidified environment. After 24 h, the cells were exposed to 0 to 4.0 mM AuNPs for 24 h. After this period the cells were trypsinised and an aliquot of the resuspended cells were counted using a haemocytomteter under a light microscope. The rest of the resuspended cells were diluted in 1% HCl solution. A known gold standard (TraceCERT^®^, Sigma-Aldrich, St Louis, MO, USA) were used to prepare a calibration curve for quantitative measurements by ICP MS. The amount of Au was calculated from the calibration curve, and uptake was expressed as pg/cell. 

### 4.2. Cell Culture

Human prostate cancer (DU145: human epithelial cells- ATCC^®^ HTB-81TM) purchased from ATCC (Manassas, VA, USA) and lung cancer (A549: human epithelial cells- ATCC^®^ CCL-185TM) cells, kindly donated by Laboratory Medicine Department, RMIT University, were used in this study.

DU145 cells were cultured and maintained in MEM Alpha + GlutaMAX^TM^ and 15 mM HEPES (Gibco^®^, Grand Island, NY, USA), 10% foetal bovine serum (FBS) (Gibco^®^) and 1% Penicillin-Streptomycin (Gibco^®^). A549 cells were cultured and maintained in DMEM/F12 supplemented with L-Glutamine and 15 mM HEPES (Gibco^®^), 10% FBS (Gibco^®^) and 1% Penicillin-Streptomycin (Gibco^®^).

Both cell lines initially were cultured and grown to 80% confluence in a 25 cm^2^ flask and then were sub-cultured. Incubation conditions during the experiments was 37 °C with 5% CO_2_ in a humidified environment.

### 4.3. AuNPs Preparation

Spherical AuNPs were purchased from Nanoprobes (Yaphank, NY, USA). This solution does not have toxic or damaging effects on biological systems and consists of AuNPs having a metal core diameter of ~15 nm, stabilized with a highly water-soluble thiol-based ligand [31]. The AuNPs used in this study was stable in both culture media i.e., MEM Alpha and DMEM/F12 and no aggregation were observed within 48 h. AuNPs cell uptake is found to be size-dependent and particles with size of 20 nm showed the highest uptake in human pancreas (PK-1, PK-45 and Panc-1) [32] and prostate (DU145) [33] cancer cells which led us to choose 15 nm AuNPs for our study as it is the closest (commercially available) size gold nanoparticles to 20 nm. In addition, 15 nm AuNPs has been reported to be non-toxic even when it is used in high concentrations [21] which differed to that seen in this study (Figure 2).

The AuNPs solution was diluted using cell culture medium to create the final concentration of 0.197 mg/mL. 

### 4.4. Viability Assay

DU145 and A549 cells (3 × 10^3^ cells/well) were seeded in a 96 well plate incubated at 37 °C with 5% CO_2_ in a humidified environment. After 24 h the cells were treated with various concentrations of AuNPs ranging from 0 to 4.0 mM and/or exposed to 0 to 6 Gy of 6 MV X-rays.

The MTS assay was performed using the CellTiter 96^®^ AQueous One Solution Cell Proliferation Assay (Promega Corp., Madison, WI, USA). At 23 and/or 47 h after treating the cells with either AuNPs or IR, the medium was removed and 100 µL of culture medium containing 20 µL of CellTiter 96^®^ AQueous One Solution Cell Proliferation Assay, was added to the cells. Immediately after adding MTS the optical absorbance of the formazan was measured at 490 nm using a CLARIOstar^®^ microplate reader (BMG Labtech, Mornington, Australia) to determine the background (BG) absorbance. The plates were then incubated for one hour followed by measuring the optical absorbance. The results are expressed as a percentage relative to the control groups as calculated in Equation (1) below:

(1) $$\mathrm{Viability}\;\%=\frac{\mathrm{Absorbance}\;\mathrm{of}\;\mathrm{treated}\;\mathrm{cells}\;-\mathrm{BG}}{\mathrm{Absorbance}\;\mathrm{of}\;\mathrm{untreated}\;\mathrm{control}\;\mathrm{cells}\;-\mathrm{BG}}\;\times \;100$$

The cell viability was measured at 24 and 48 h after treatment with AuNPs and/or IR.

### 4.5. Cell Irradiation

Cells were irradiated with 6 MV X-ray generated by Linac (Elektra Synergy, Stockholm, Sweden) located at Australian Radiation Protection and Nuclear Safety Agency (ARPANSA), Yallambie, Australia). The radiation was delivered as a single fraction for each dose i.e., 0 Gy for control groups and 2 or 5 Gy for treated groups.

### 4.6. Irradiation Setup

**Figure 11:**
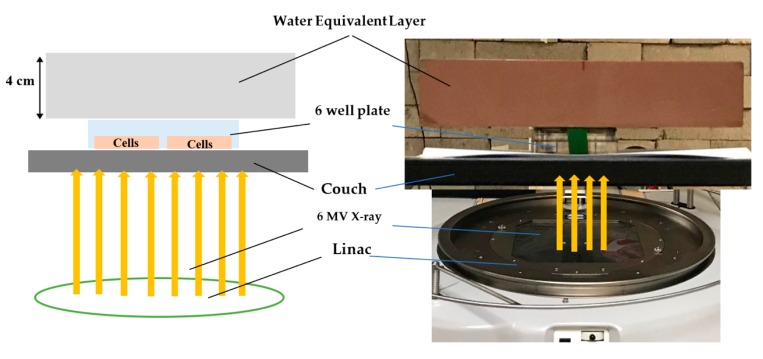
Cell irradiation setup.

Since the cell lines used for this study are adherent, a monolayer of cancer cells is formed at the bottom of 6-well plates, the experimental plates were uniformly exposed in bottom to top direction. A water equivalent layer with the thickness of 4 cm placed on top of the plates, provided sufficient backscatter radiation to form an electric equilibrium as seen in Figure 11.

### 4.7. Scratch Assay

DU145 and A549 cell lines were cultured in 6 well plates for 24 h until they reached 80–90% confluence. A gap of size of ~600 µm was created using a sterile (200 μL) yellow pipette tip and the tissue culture media were replaced with fresh media and CytoSmart^®^ Live Image System (Piet Heinstraat, Zutphen, Holland) was used to observe the gap-filling process. The images were taken every 5 min in a 5% CO_2_ incubator maintained at 37 °C. The gap area at 0 h and 24 h were measured with ImageJ^®^ software and the relative migration was calculated using Equation (2) as seen below.

(2) $$\mathrm{Relative}\;\mathrm{Migration}=\frac{\mathrm{Gap}\;\mathrm{area}\;\mathrm{at}\;0\;h-\mathrm{Gap}\;\mathrm{area}\;\mathrm{at}\;24\;h\;\left(\mathrm{Untrated}\;\mathrm{control}\;\mathrm{cells}\right)}{\mathrm{Gap}\;\mathrm{area}\;\mathrm{at}\;0\;h-\mathrm{Gap}\;\mathrm{area}\;\mathrm{at}\;24\;h\;\left(\mathrm{Treated}\;\mathrm{cells}\right)}$$

To evaluate the changes of relative migration over time and estimate the gap filling rate for untreated control and treated groups, relative migration was measured in every 2 h and the results plotted as a graph of relative migration vs. time. The slope of each graph was considered as the gap filling rate as tabulated in Table 1.

### 4.8. Adhesion Assay

The cell’s adhesion to the polystyrene surface of the well of a 6-well plate was evaluated in both control and treated groups. Cells were exposed to 2 and/or 5 Gy 6 MV X-ray and/or treated with 1 mM AuNPs, in 25 cm^2^ flasks and were incubated for 24 h. Then each experimental group i.e., control (untreated), irradiated with 2 or 5 Gy and/or treated with AuNPs were trypsinised and 2 × 10^4^ cells seeded in 6 well plates. After 4 h incubation at 37 °C, the non-adherent cells were gently washed off with warm PBS (37 °C) and images were taken using an EVOS^®^ XL Cell Imaging System (Thermo Fisher Scientific, Waltham, USA). The adherent cells were determined by counting the attached cells to a 6-well plate in a 6.25 × 10^4^ μm^2^ and for each well pate four different random areas were counted.

### 4.9. Statistical Analysis

All presented data within this paper are the mean of at least three independent experiments. Statistical comparison between control group and IR group, AuNPs group, and IR + AuNPs group were performed using one-way analysis of variance (ANOVA) with IBM SPSS Statistics version 25 (IBM Australia Ltd., NSW, Australia). Results are reported as mean ± standard error of the mean (SEM). * *p* < 0.05 were considered statistically significant.

## Figures and Tables

**Table 1:** Effect of IR and/or AuNPs on the gap-filling rate in prostate (DU145) and Lung (A549) cancer cells. Results expressed as the mean for 3 replicates. Significance of gap closure between 0–6 h compared to 8–24 h is shown as * *p* < 0.05.

	Experimental Groups	Gap Filling Rate % Change/h 0–6 h	Gap Filling Rate % Change/h 8–24 h	
**Prostate Cancer** **DU145**	Control	−0.050	−0.033	* (***p*** < 0.05)
	IR	−0.032	−0.032	-
	AuNPs	−0.027	−0.026	-
	IR + AuNPs	−0.015	−0.021	-
**Lung Cancer** **A549**	Control	−0.053	−0.021	* (***p*** < 0.05)
	IR	−0.032	−0.024	-
	AuNPs	−0.030	−0.020	-
	IR + AuNPs	−0.015	−0.020	-

## Abbreviations

IR	ionising radiation
LET	lineal energy transfer
MV	mega voltage
EMT	epithelial-mesenchymal transition
MMP	matrix metalloproteinase
ECM	extracellular matrix
NPs	nanoparticles
AuNPs	gold nanoparticles
AuNRs	gold nanorads
Au@Pt-Nss	gold-platinum nanoseeds
PTC	papillary thyroid cancer
SEM	standard error of mean
ROS	reactive oxygen species
FBS	foetal bovine serum
BG	background
ICP-MS	inductively coupled plasma-mass spectrometry
